# Intervillous Macrophage Migration Inhibitory Factor Is Associated with Adverse Birth Outcomes in a Study Population in Central India

**DOI:** 10.1371/journal.pone.0051678

**Published:** 2012-12-13

**Authors:** Puspendra P. Singh, Naomi W. Lucchi, Anna Blackstock, Venkatachalam Udhayakumar, Neeru Singh

**Affiliations:** 1 Regional Medical Research Center for Tribals, Garha, Jabalpur Madhya Pradesh, India; 2 National Institute of Malaria Research Field Unit, Jabalpur, Madhya Pradesh, India; 3 Malaria Branch, Division of Parasitic Diseases and Malaria, Center for Global Health, Centers for Disease Control and Prevention, Atlanta, Georgia, United States of America; 4 Atlanta Research and Education Foundation/VA Medical Center, Decatur, Georgia, United States of America; Royal Tropical Institute, The Netherlands

## Abstract

Macrophage migration inhibitory factor (MIF) is a pluripotent factor produced by a variety of cells. It plays an important biological role in the regulation of pregnancy and has been shown to influence malaria pathogenesis. In this study, the levels of MIF in the peripheral, cord and placental intervillous blood (IVB) plasma collected from women residing in a malaria endemic region of Central India was determined and its association with malaria in pregnancy and birth outcomes was investigated. MIF levels were significantly different in IVB, peripheral, and cord plasma, with IVB plasma having the highest MIF levels and peripheral plasma having the lowest. Placental malaria positive women had significantly higher IVB plasma MIF levels than placental malaria negative women, but this relationship was not seen in peripheral or cord plasma MIF levels. In addition, the odds of stillbirth and low birth weight deliveries for the uppermost placental MIF quartile (irrespective of placental malaria status) was significantly higher than that of the lowest placental MIF quartile, supporting the hypothesis that elevated concentrations of placental MIF may be associated with an increased risk of adverse birth outcome.

## Introduction

A hallmark of malaria during pregnancy is the sequestration of malaria-infected red blood cells (iRBCs) containing late developmental stages of malaria parasite such as late trophozoite and early schizonts in the intervillous spaces (IVS) of the placenta [1], [2]. This is usually accompanied by the infiltration of maternal leukocytes, especially monocytes, in the IVS [3], [4] and hemozoin deposition [1], [5], resulting in what is referred to as placental malaria (PM). PM poses substantial risk for poor fetal outcomes such as low birth weight (LBW), prematurity and stillbirths, as reviewed in [6]. A lot of what is known about malaria in pregnancy comes from studies conducted in Africa. Although the prevalence of malaria in pregnant Indian women is lower than the prevalence in African women [7], [8], several epidemiological studies have demonstrated that malaria in pregnancy is a public health problem in India as well [8]–[14], [15], [16]. In India, commonly observed complications due to malaria in pregnancy include many of the same complications observed in African settings, namely preterm deliveries, intra uterine death, LBW, stillbirths and abortions [16].

The immunological consequences of malaria in pregnancy have been widely investigated. Several studies have demonstrated the presence of both proinflammatory and anti-inflammatory immune factors in malaria-infected placentas [17]–[23]. However, while proinflammatory responses are important for the clearance of iRBCs and for the protection against PM, they have also been shown to play a role in malaria pathophysiology and to contribute to morbidity associated with malaria in pregnancy [24]. These proinflammatory immune responses have been associated with the ensuing monocytic infiltration [3], [21] which has been associated with poor fetal outcomes [3], [4], [25]. Of interest in pregnancy and pregnancy-associated infections is the immune factor macrophage migration inhibitory factor (MIF), a proinflammatory cytokine involved in physiological and pathological processes in pregnancy. MIF is a pluripotent cytokine produced by a variety of normal cell types and tissues, including macrophages, monocytes, endocrine glands and reproductive organs [26]. MIF is capable of inhibiting the random migration of mononuclear cells and of circumventing the effects of glucocorticoid-mediated immunosuppression often observed in pregnancy. MIF was shown to be highly elevated in the placenta of pregnant women with malaria [23], [27] and other intrauterine infections [28] and in peripheral serum of women with preeclampsia [29], [30]. While MIF plays a major role in normal pregnancy, delivery and even labor [31], [32], increased levels of amniotic fluid MIF [28] and peripheral serum MIF [33] were shown to be associated with preterm labor. Given that elevated MIF levels have been observed in women with PM, determining whether or not MIF contributes to the poor fetal outcomes associated with PM is of interest.

In this study, we investigated the relationship between MIF levels in IVB, peripheral, and cord plasma samples in pregnant women residing in a low transmission setting in Central India. We also compared MIF levels for PM+ and PM- women and investigated the relationship between MIF and adverse outcomes associated with *P. falciparum* infection.

## Materials and Methods

### Study Site and Sampling Method

**Figure 1:**
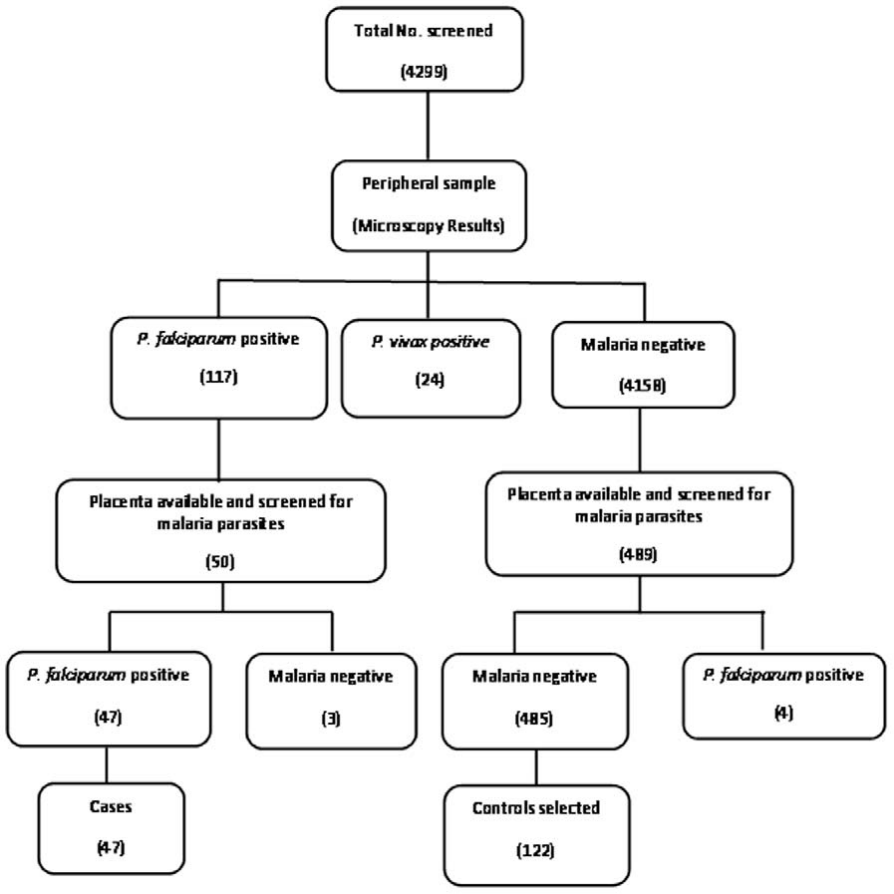
Schematic flow chart summarizing the sampling process. A total of 4299 women were screened for this study. Forty seven women positive for *P. falciparum* infection in both placental and peripheral blood by microscopy were enrolled into the study (PM+ cases). Out of the 485 women who had no malaria parasite in the peripheral as well as placental smear, 122 were selected and included as healthy controls (PM-group).

This study was carried out between July 2008 and December 2009 in the delivery unit at Civil Hospital, Maihar, India. This is a secondary health facility that caters to the health needs of rural, semi-urban and ethnic tribal population of Central India. This study was approved by the ethical research committee of Regional Medical Research Center for Tribals, Jabalpur, India, and the Centers for Disease Control and Prevention, Atlanta, GA, USA. Pregnant women who presented at the delivery unit were screened for malaria by microscopic examination of a peripheral blood smear. Figure 1 summarizes the sampling process in this study. A total of 4299 women were screened, and women over the age of 18 with no chronic disease were invited to participate in the study. By microscopy, 47 women were found to have *P. falciparum* infection in both placental and peripheral blood (Figure 1), and these were included in the study as the placental malaria positive cases (PM+). All 485 women whose peripheral and placental smears were malaria negative could not be enrolled in the study due to budgetary constraints, so a subset of 122 was selected to be in the placental malaria negative (PM- control group). The PM- group was chosen so that it would have approximately the same percentage of LBW babies, the same percentage of stillbirths, and the same gravidity distribution as the PM+ women. The purpose of the study was described to all the participants who were enrolled only after obtaining written consent. All consent forms were kept at Regional Medical Research Centre for Tribals, Jabalpur, India.

### Sample Collection

Upon enrollment, socioeconomic status, fever history, clinical symptoms, hemoglobin and auxiliary temperature were recorded for each woman. Peripheral blood smears (thick and thin) were taken for malaria parasite examination and about 1 ml of peripheral blood was collected for immunological study. After delivery, the expelled placenta was collected and processed for intervillous blood (IVB) and cord blood collection. The IVB was collected using the incision method [1]; two to three shallow cuts approximately 2 mm deep were made into the maternal surface of the placenta’s third quadrant using a sterile scalpel. About one ml of placental blood that seeped into these incisions was collected using a blunt 1-ml syringe and transferred into a heparinized microcentrifuge tube. Cord blood was taken by manual compression of the umbilical cord. Subsequently, placental blood smear, tissue impression smear and cord blood smear were prepared for malaria parasite examination. Plasma was prepared from peripheral, IVB and cord blood and was stored in −80°C until used for experiments. The blood films were stained with Giemsa and examined by an experienced technician in the field laboratory using an oil-immersion lens (100×magnification). The technician examined 100 microscopic field of thick smear before declaring a smear as negative [34]. All blood films were read twice by different microscopists at a reference laboratory. Discordant results were given a third reading by a microscopist blinded to the results of the first two readings, the result of which was considered as final. Histological examination of the placenta was not performed.

### Birth Outcomes

A baby born with no sign of life at or after 28 weeks of gestation was considered a stillbirth. Live birth was defined as complete expulsion of a live baby, irrespective of the duration of the pregnancy. Babies with birth weight below 2500 grams were considered LBW babies, and other babies were considered to have normal birth weight (NBW). Note that birth weight was not measured for stillbirths.

### MIF Level Estimation

The levels of MIF in IVB, peripheral and cord blood plasma were measured by a standard enzyme-linked immunosorbent assay (ELISA) using a commercially available kit (R&D Duoset, Minneapolis, MN) as previously describes [23].

### Statistical Analysis

Mother characteristics and birth outcomes were compared for PM+ and PM− women. Chi-square tests were used to compare PM groups in terms of categorical variables, and t-tests were used for continuous variables since these variables did not strongly deviate from normality. Since MIF levels were highly skewed, the log MIF levels were used in linear models with MIF as the outcome. Log MIF levels for IVB, peripheral, and cord plasma samples were compared using a linear mixed model, allowing measurements from the same mother to be correlated. Another linear mixed model was used to compare log MIF levels of PM+ and PM− women. A PM status by plasma type (IVB, peripheral, or cord) interaction was included initially so that it could be determined whether or not the difference in log MIF level varied by plasma type. Like the model for plasma type, this model allowed measurements from the same mother to be correlated. Hemoglobin level, maternal age, auxiliary temperature, plasma type, and gravidity were controlled for in the PM model.

Logistic regression was used to investigate the relationship between IVB plasma MIF levels and birth outcomes. Since odds ratios for birth outcomes did not appear to have a linear association with log MIF levels, quartiles were used to define MIF categories, and odds ratios were calculated for the three upper quartiles using the lowest quartile as the reference. The model for the stillbirth outcome controlled for PM status, hemoglobin level, maternal age, auxiliary temperature, and gravidity. For the LBW outcome, stillbirths were excluded, and the model controlled for PM status, hemoglobin level, maternal age, auxiliary temperature, gestational age, and gravidity. All statistical analyses were performed using SAS v. 9.3 (SAS Institute Inc., Cary, NC, USA).

## Results

### Patient Characteristics

A total of 169 women (47 PM+ and 122 PM−) were enrolled into the study. Of these, 63 were primigravidae (first pregnancy), 30 secundigravidae (second pregnancy) and 76 were multigravidae (more than two previous pregnancies). Since PM− and PM+ groups were matched in terms of birth weight, stillbirths, and gravidity, these variables did not and were not expected to differ between the two groups. As summarized in Table 1, PM+ women had significantly higher body temperature and were more anemic than PM− women.

**Table 1:** Mother characteristics and birth outcomes by placental malaria status.

	Malaria Positive†	Malaria Negative†	P Value**
	(N = 47)	(N = 122)	
**Characteristics of Mothers**			
Average age (years)	25.2±3.9	25.1±3.9	0.87
Auxiliary Temperature (°C )	37.0±1.1	35.9±1.4	<0.001
Mean Hemoglobin (g/dl)	10.1±2.1	11.5±2.4	<0.001
Anemia % (<11 g/dl)	32 (68%)	49 (40%)	0.001
**Gravidity** *			
Primigravid	22 (47%)	41 (34%)	0.25
Secundigravid	6 (13%)	24 (20%)	
Multigravid	19 (40%)	57 (47%)	
**Log MIF**			
IVB Plasma	8.43±0.95	7.97±0.78	0.001
Peripheral Plasma‡	2.76±1.21	2.37±0.84	0.051
Cord Plasma‡	5.46±1.16	5.03±1.30	0.22
**Birth Outcomes**			
Baby Birth Weight (g) ‡	2324.1±452.9	2440.4±413.2	0.16
Low Birth Weight (n)* ‡	22 (59%)	56 (57%)	0.76
Stillbirth (n)*	10 (21%)	23 (19%)	0.72

* Indicates variable used to frequency match PM+ and PM− groups.

** P-values reported are from Pearson chi-square test for categorical variables and two-sample t-test for continuous variables.

† Numbers reported are mean ± standard deviation for continuous variables and n (%) for categorical variables.

‡ Birth weight information was collected for 37 babies born to PM+ women and 99 babies born to PM− women (birth weight was not collected for stillbirths).

Peripheral plasma samples were available for 47 PM+ women and all 122 PM− women.

Cord plasma samples were available for 20 PM+ women 43 PM− women.

Note that all PM+ and PM− women had IVB plasma samples available.

### MIF Level in IVB, Cord and Peripheral Plasma

**Figure 2:**
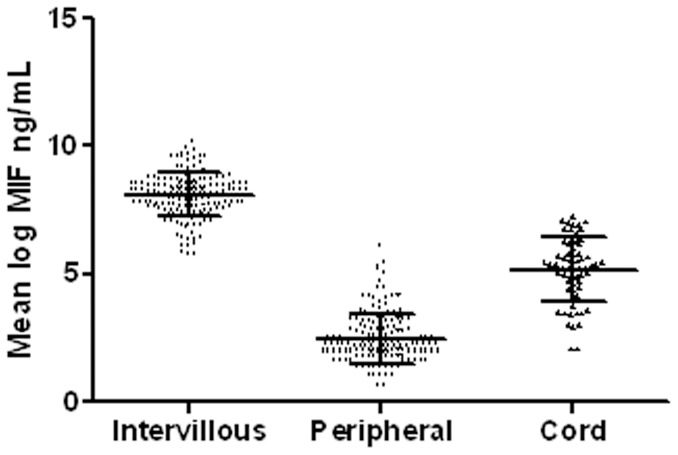
Elevated MIF levels observed in IVB plasma. The IVB, peripheral, and cord plasma log MIF levels were significantly different from one another (p<0.001). Horizontal bars indicate mean of log MIF +/− SD.

MIF was detected in 169 IVB samples, 167 of the peripheral samples and in 63 of the cord samples. Several reasons contributed to the missing cord samples: stillbirth deliveries (24 cases), caesarean delivery (34 cases), retained placenta (22 cases), post partum haemorrhage (13 cases) and delayed collection of placenta (13 cases). As shown in Figure 2, means of log MIF levels were significantly different for the three plasma types (p<0.001 for all pairwise comparisons), with IVB plasma having the highest log MIF level, followed by cord and peripheral samples.

### Elevated IVB MIF in PM Positive Women

Analysis revealed that the mean log MIF level was significantly higher in PM+ women compared to PM− women when controlling for possible confounders (Table 1) (difference of 0.39; CI 0.16–0.63; p = 0.001). There was no significant difference in the peripheral and cord MIF levels based on infection malaria infection status.

### Effects of MIF on Delivery Outcomes

For the stillbirth outcome, the odds ratio for the uppermost MIF quartile versus the lowest MIF quartile was found to be significant, meaning that the odds of having a low birth weight baby was elevated in the highest MIF category when compared to the lowest MIF category (OR 5.06; 95% CI 1.65 to 15.53; p = 0.005). The odds of the adverse birth outcomes did not differ for the other two MIF quartiles when compared to the lowest MIF quartile. In the model for LBW, a similar relationship was seen between the uppermost MIF quartile and the lowest (OR 9.09; 95% CI 1.96 to 42.21; p = 0.005) (Table 2). Overall, these associations were evident irrespective of placental malaria infection status.

**Table 2:** Association of IVB log MIF levels and poor birth outcomes.

MIF Quartile**	Stillbirth*(N = 169)				LBW† (N = 136 )			
	Proportion	OR	95% CI	P Value	Proportion	OR	95% CI	P Value
1	7/43 (16%)	1	–	–	17/36 (47%)	1	–	–
2	4/42 (10%)	0.66	(0.19–2.32)	0.52	23/38 (61%)	2.24	(0.71–7.05)	0.18
3	4/42 (10%)	0.61	(0.17–2.18)	0.45	19/38 (50%)	1.14	(0.35–3.75)	0.53
4	18/42 (43%)	5.06	(1.65–15.53)	0.005	19/24 (79%)	9.09	(1.96–42.21)	0.005

* Stillbirth model controls for placental malaria status, hemoglobin level, maternal age, auxiliary temperature, and number of pregnancies (primigravid, secundigravid, or multigravid).

** Cutoff values for MIF quartiles are 2078, 3554, and 5283, corresponding to log MIF values of 7.64, 8.18, and 8.57.

† LBW model is for live births only and controls for placental malaria status, hemoglobin level, maternal age, auxiliary temperature, gestational age, and number of pregnancies (primigravid, secundigravid, or multigravid).

## Discussion

A previous study by Chaisavaneeyakorn *et al*. reported significantly elevated MIF levels in the IVB plasma of Kenyan pregnant women compared to both peripheral and cord plasma [23]. Similar results were obtained by Chaiyaroj *et al*., who observed significantly higher MIF production by intervillous blood mononuclear cells (IVBMC) compared to peripheral blood mononuclear cells (PBMC), as well as high MIF level in placental plasma compared to paired peripheral plasma [27]. Our finding confirms this observation in an Indian population as MIF was shown to be present at very high levels in the IVB plasma as compared with peripheral and cord plasma. This finding is not surprising given that MIF has been shown to play important roles during normal pregnancy [31], [32] and therefore, IVB MIF would be expected to be high.

We observed significantly higher IVB MIF levels in women with PM compared to PM− women. Similar findings were reported by Chaisavaneeyakorn *et al*. who suggested that MIF may play a role in the pathogenesis of malaria [23]. MIF is a pluripotent immune factor known to regulate both innate and adaptive immune responses to bacterial and parasitic infections. Several studies have demonstrated elevated levels of MIF in the peripheral or placental blood during malaria infections [23], [35]–[37]. MIF was shown to be induced in response to *P. chabaudi* infection in mice and was implicated in the pathogenesis of malaria anemia [35]. Awandare *et al.* suggested that reduced MIF production may promote enhanced disease severity in children with *P. falciparum* malaria [36], implying that MIF may be required to clear a malaria infection. One of the roles of MIF is to inhibit the random migration of macrophages which has been demonstrated in vitro [38]. We hypothesize that the increased MIF levels found in the placenta of malaria positive women is induced by the malaria parasites which tend to accumulate in the placenta [3], [21] and that this may help to retain macrophages in the placenta. MIF, together with other proinflammatory factors such as IFN-γ and TNF-α, may then activate these macrophages to clear and kill malaria parasites in the placenta.

It is generally accepted that about 50% or more of stillbirths in low and middle income countries are caused by maternal intrauterine infections (reviewed in [39]). It has also been suggested that excessive proinflammatory immune responses may lead to adverse placental pathophysiology. These proinflammatory immune responses have been associated with the ensuing monocytic infiltration [3], [21] observed in malaria infected placentas and which has been associated with poor fetal outcomes such as LBW [3], [4], [25]. In this study, higher levels of IVB MIF were associated with stillbirth deliveries and LBW babies irrespective of malaria infection status. To our knowledge, this is one of the first reports to demonstrate an association between elevated placental MIF levels and poor birth outcomes. Previous studies have shown that maternal peripheral serum MIF is associated with preterm delivery [40] and preeclampsia [30]. Two different explanations can be considered for the current findings. One possibility is that elevated levels of MIF may directly regulate some biological events related to stillbirth and LBW. For example, high levels of MIF (along with other factors such as TNF) can activate the inducible cyclooxygenase (COX)-2 pathway, leading to higher prostaglandin production and potentially other pathways that contribute to stillbirth and LBW. MIF was shown to directly stimulate the synthesis of COX-2 and the release of prostaglandin E_2_ in ectopic endometrial cells [41]. Increased levels of prostaglandin E_2_ and prostaglandin F_2α_ induced by alcohol were shown to cause fetal death [42]. Therefore, MIF may have a direct role in activating biological pathways that contribute to stillbirths and LBW. The second possibility is that elevated levels of MIF may be simply a marker for proinflammatory events occurring in the IVB and therefore, simply serve as a predictor of the events leading to the poor outcomes. A proinflammatory milieu is commonly observed in malaria infected placentas and this has been associated with poor fetal outcomes such as LBW [3], [4], [25]. It is not surprising that this association was evident irrespective of placental malaria infection status because the elevated levels of MIF can occur due to many infectious agents and once MIF is activated beyond a particular threshold it is likely to trigger biological pathways that contribute to poor birth outcomes.

LBW is a hallmark of malaria during pregnancy with several studies demonstrating an increase in LBW babies delivered by PM+ women compared to PM- women. [3], [4], [25], [43], [44]. A caveat of this study is the fact that we could not test for any differences in birth weight in these two groups since the percentage of low birth weight of infants born to PM− women was matched to that of PM+ women in the selection process. Therefore, the apparent lack of differences in birth weight in this study (mean birth weight <2500 g) is due to an enrolment artefact and might not have any generalizable bearing to this population.

In conclusion, this study demonstrates that intervillous MIF is significantly elevated in the presence of placental malaria. In addition, elevated intervillous MIF levels are associated with both stillbirth and low birth weight deliveries.
